# Risk factors contributing to the incidence and mortality of acute childhood poisoning in emergency department patients in Iran: a hospital-based case-control study

**DOI:** 10.4178/epih.e2019016

**Published:** 2019-04-23

**Authors:** Hamideh Feiz Disfani, Mostafa Kamandi, Seyed Mohammad Mousavi, Sayyed Majid Sadrzadeh, Roohie Farzaneh, Najme Doolabi, Kazem Rahmani

**Affiliations:** 1 Department of Emergency Medicine, Faculty of Medicine, Mashhad University of Medical Sciences, Mashhad, Iran; 2 Department of Internal Medicine, Faculty of Medicine, Mashhad University of Medical Sciences, Mashhad, Iran; 3 Department of Epidemiology, School of Public Health, Iran University of Medical Sciences, Tehran, Iran

**Keywords:** Poisoning, Child, Mortality, Emergency department, Iran

## Abstract

**OBJECTIVES:**

Since poisoning is one of the most important preventable factors contributing to the hospitalization and death of children who present to emergency departments, this study was carried out to investigate the risk factors contributing to the incidence and mortality of acute childhood poisoning.

**METHODS:**

This hospital-based case-control study included 243 cases and 489 controls, drawn from daily admissions to the emergency departments of the included hospitals according to the inclusion and exclusion criteria.

**RESULTS:**

Gastrointestinal poisoning was the most common poisoning type, found in 87.7% of subjects, and medications were the most common cause of poisoning (49.8%). Multiple logistic regression analysis showed that a history of poisoning (odds ratio [OR], 10.44; 95% confidence interval [CI], 5.58 to 19.51; p<0.001) and the availability of poisonous substances (OR, 8.88; 95% CI, 5.41 to 14.56; p<0.001) were among the most important predictors of childhood poisoning. Respiratory poisoning (OR, 6.72; 95% CI, 1.40 to 32.07; p<0.05) and the presence of addiction in the family (OR, 4.54; 95% CI, 1.10 to 18.68; p<0.05) were the most important predictors of mortality among children with poisoning.

**CONCLUSIONS:**

Addiction and the presence of physical or psychological disorders in family members, a history of poisoning, and the availability of poisonous substances were significantly associated with the incidence of childhood poisoning and resultant mortality.

## INTRODUCTION

Poisoning is a major public health problem and a significant cause of mortality and morbidity in children, accounting for approximately 3.0% of all patients admitted to emergency departments (EDs) [1-3]. Every year, approximately 45,000 children and adolescents under the age of 20 die from poisoning, and it has been reported that the death rate from poisoning in people under the age of 20 is 1.8 per 100,000 worldwide [4,5].

Studies have suggested that the mortality rate from poisoning varies according to the cultural and geographical characteristics of different communities [6-8]. In developed countries, the mortality rate is roughly 0.5 per 100,000 people, while it is dramatically higher in low-income and underdeveloped countries, reaching about 2.0 per 100,000 people (almost 4 times higher than the rate in high-income and developed countries) [9,10]. Approximately 80% of all poisoning cases occur between the ages of 1 year and 5 years, and the most common cause of poisoning in children younger than 1 year old is medication administered by parents to children [6,11,12].

The main causes of poisoning in children include the use of various drugs, exposure to hydrocarbons such as petroleum products, bleaching agents, detergents and disinfectants, plant pesticides, insecticides, and the use of cosmetics, alcohol, and narcotics; however, more than 75% of poisoning cases occur as a result of consuming poisonous substances [13,14]. It is also well established that increased access to and use of chemicals for various purposes such as medicine, agriculture, and industry, accompanied by changes in individuals’ lifestyle, social behaviors, and economic factors, have led to increases in the rate of poisoning and mortality due to poisoning throughout the world [13,15,16].

Cases of poisoning not only cause major damage to communities’ health and economic well-being, but also place a tremendous psychological and emotional burden on families, which must be taken into consideration when assessing the scale of this problem [5,8]. Identifying the risk factors for poisoning is an essential measure for poisoning prevention programs in any society, and the rate of poisoning and resultant mortality can be reduced by modifying community-level risk factors [10].

In this regard, given the impact of factors related to acute childhood poisoning and the lack of sufficient evidence on the epidemiology of these factors in Iran, the present study aimed to identify risk factors for acute childhood poisoning, focusing both on incidence and mortality.

## MATERIALS AND METHODS

This hospital-based case-control study was carried out after obtaining permission from the organizational Ethics Committee of Mashhad University of Medical Sciences. This study included patients admitted to the EDs of 2 large hospitals (Imam Reza and Ghaem) in Mashhad, Iran in 2018. All children under the age of 15 years admitted to the ED on a daily basis were included in the study based on the inclusion and exclusion criteria and after informed consent was obtained from their parents. The exclusion criteria were unwillingness of the parents to participate, childhood cognitive impairment and congenital genetic diseases, neurological and metabolic diseases, brain infections and trauma of the central and peripheral nervous systems, and physical-motor disabilities. Patients with some degree of respiratory or gastrointestinal poisoning were selected through the census sampling method and enrolled in the study based on the daily admissions records of the EDs of the abovementioned hospitals. The control group comprised children admitted to the emergency and related departments (e.g., the orthopedic department, the internal medicine department, and the burn ward) for reasons other than poisoning, who were randomly selected from the hospital records and enrolled in the study. The individuals in both the case and control groups were selected daily, separately, and under the supervision of an emergency medicine specialist. The intended control-to-case ratio was 2:1 (2 controls per 1 case). After admission, the patients were transferred from the ED to the appropriate wards for hospitalization and further treatment.

To investigate the risk factors for childhood poisoning, checklists were used that included demographic, behavioral, and socioeconomic factors possibly affecting the risk of childhood poisoning, including the mother’s employment, parental education, parental smoking, previous history of poisoning, addiction in the family, income, the presence of physical or mental illness in the family, and family size. The duration of hospitalization, the type of poisoning, and eventual clinical outcomes were also examined.

### Statistical methods

Descriptive statistics, such as the mean and standard deviation, were used to present the data. The odds ratios (ORs) and 95% confidence intervals (CIs) of the risk factors associated with the incidence of childhood poisoning were calculated using a univariate logistic regression model. In order to eliminate the effect of confounding factors, variables with a significance level of p-value<0.1 in the univariate logistic regression model were introduced into a multivariate logistic regression model [17,18]. Receiver operating characteristic (ROC) analysis and the area under the ROC curve were used to assess the discriminative ability of the multiple logistic regression model. The statistical analysis was carried out using Stata version 12 (StataCorp., College Station, TX, USA), and the significance level in this study was p-value<0.05.

## RESULTS

In this study, 243 patients comprised the case group and 489 comprised the control group. In the case group, 108 patients (44.4%) were aged 1-4 years, and 16.0% of the children with poisoning were younger than 1 year old (Table 1). Most of the poisoned children (87.7%) had gastrointestinal poisoning, and the remaining 12.3% of poisoning cases had respiratory poisoning. The main causes of poisoning were medications (49.8%), detergents and disinfectants (16.5%), carbon monoxide (12.3%), chemicals and petroleum (12.3%) and others (9.0%), including drugs, alcohol, and food. Furthermore, 37.5% of the cases (n=91) had a history of poisoning, 30.4% of the cases (n=75) had hyperactivity, and 31.3% of the families with poisoned children had at least 1 addicted member. Finally, 96.2% of the patients in the case group were treated or discharged, and the specific mortality rate from poisoning was 3.8%. Other information about the individuals studied is shown in Table 1.

The univariate logistic regression model (Table 2) of risk factors for childhood poisoning showed that the age groups of 1-4 years old (OR, 2.20; 95% CI, 1.43 to 3.38; p<0.001) and <1 year old (OR, 8.09; 95% CI, 4.17 to 15.71; p<0.001) were at the highest risk of poisoning. The mother’s occupation also affected the incidence of childhood poisoning, with the children of employed mothers having a higher risk of poisoning (OR, 2.80; 95% CI, 2.01 to 3.89; p<0.001). Children’s hyperactivity was a significant independent factor that increased the risk of childhood poisoning (OR, 3.25; 95% CI, 2.20 to 4.79; p<0.001). Other factors significantly associated with an increased risk of poisoning in children (p<0.01) included a previous history of poisoning, addiction in the family, accessibility of poisonous substances, urban residence, and the presence of physical diseases or psychological disorders in family members.

The multiple logistic regression analysis (Table 2) showed that the following risk factors had a significant relationship with the incidence of childhood poisoning (p<0.05): age, mother’s occupation, mother’s smoking status, history of poisoning, family history of addiction, child hyperactivity, accessibility of poisonous substances, and the presence of physical diseases or psychological disorders in family members. A history of poisoning (OR, 10.44; 95% CI, 5.58 to 19.51; p<0.001), accessibility of poisonous substances (OR, 8.88; 95% CI, 5.41 to 14.56;p<0.001), and being <1 year old (OR, 6.62; 95% CI, 2.95 to 15.00; p<0.001) were the most important factors influencing the incidence of childhood poisoning.

**Figure 1.**
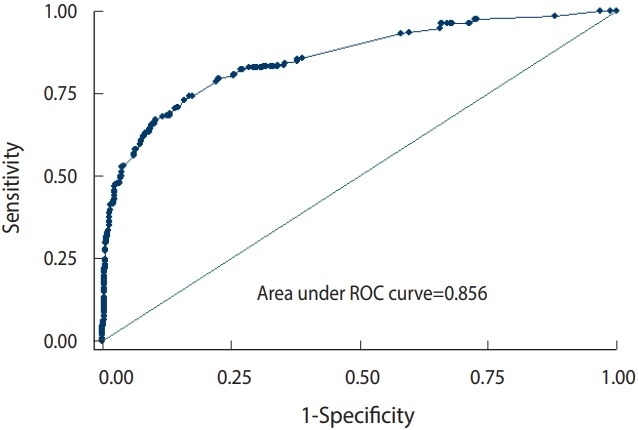
Evaluation of the multiple logistic regression model through the area under the receiver operating characteristic (ROC) curve.

In the assessment of the model, the area under the ROC curve was 0.856, which clearly indicated the viability of the multiple logistic regression model in this study (Figure 1).

As shown in Table 3, respiratory poisoning (OR, 6.72; 95% CI, 1.40 to 32.07; p<0.05) and a family history of addiction (OR, 4.54; 95% CI, 1.10 to 18.68; p<0.05) increased the risk of mortality in poisoned children. Other factors, such as mother’s occupation, mother’s smoking status, hyperactivity, accessibility of poisonous substances, a previous history of poisoning, and the presence of physical diseases or psychological disorders in family members did not have a significant relationship with mortality in poisoned children.

## DISCUSSION

Poisoning is one of the most important preventable factors contributing to the hospitalization and death of children in EDs. The results of this study, which aimed to investigate the risk factors affecting childhood poisoning, clearly identified factors associated with the incidence and mortality of poisoning in children. Although the epidemiology of childhood poisoning shows significant geographic variation, regions with similar socioeconomic and cultural factors tend to have similar epidemiological patterns [19]. Age is one of the most important factors affecting the incidence of childhood poisoning, but the role of age varies across regions. In developing countries, the highest incidence of poisoning is in children aged <5 years, and such cases are often involuntary and out of curiosity, while in developed countries, the highest incidence of poisoning is observed in individuals over the age of 60 [19-21]. Talebian et al. [22] and Andiran & Sarikayalar [23] showed that the age group of 1-5 years had the highest frequency of poisoning. In our study, children under the age of 4 years had the highest incidence and risk of poisoning, which is consistent with the results of other studies. Lamireau et al. [24] also showed that more than 80% of childhood poisoning cases occurred in children younger than 6, while Pawłowicz et al. [3] found the age group of 16-18 to have the highest rate of poisoning. They reported that poisoning in children younger than 5 years was observed in fewer than 15% of the subjects, which is inconsistent with our study results and shows the epidemiological differences in poisoning depending on countries’ cultural and social development.

In this study, maternal factors such as smoking and occupational status were associated with the risk of poisoning in children. Studies in developed countries have concluded that the absence of at least 1 parent could increase the risk of unintentional poisoning in children [12]. Mansori et al. [18] likewise showed that maternal factors such as employment, education, and smoking status increased the risk of childhood poisoning, which is consistent with the results of our study; in this study, the risk of poisoning in children with smoking mothers was 3.36 times higher than in those with non-smoking mothers. Maternal employment also increased the risk of childhood poisoning by 2.8 times.

Children’s hyperactivity has also been identified as an important factor in the prevalence of poisoning, and even injuries and traumas, in this age group. Ruiz-Goikoetxea et al. [25] showed a clear relationship between children’s hyperactivity and poisoning. The results of this study indicated that hyperactive children were at a high risk of poisoning, which is in line with the results of previous studies. Brayden et al. [26] suggested that factors such as easy access to poisonous substances and children’s curiosity were among the most important risk factors for poisoning. Even the absence of 1 parent during the day due to their occupation, especially mothers, was associated with an increased risk of poisoning in children [12,27]. Accessibility of poisonous substances, addiction in the family, the presence of a physical or psychological illness in the family, and a history of poisoning were among the other factors that increased the risk of childhood poisoning [10,17,18]. The results of studies by Mansori et al. [18] and Dayasiri et al. [28] showed that inaccessibility of substances that might cause poisoning played a protective role against childhood poisoning. In contrast, easy access of children to poisonous substances was considered to be one of the most important factors affecting childhood poisoning. The presence of addiction among family members could increase the risk of childhood poisoning [10,18]. However, Dayasiri et al. [28] reported that drug and alcohol addiction significantly reduced the risk of childhood poisoning, which is not consistent with the results of our study. In addition, the results of studies that are consistent with ours confirmed that parents’ level of knowledge and their attitude towards poisonous substances were among the most important risk factors [10,18,29], as observed in the present study.

Addiction in the family and respiratory poisoning were associated with mortality in poisoned children in this study. Respiratory poisoning, mainly due to carbon monoxide, increased the risk of child mortality by 6.7 times compared to gastrointestinal poisoning. This underscores the important role of respiratory poisoning caused by carbon monoxide in child mortality in developing countries. Carbon monoxide poisoning has symptoms ranging from mild (e.g., headache, faint, lethargy, and myalgia) to severe (e.g., diplopia, syncope, coma, cardiac arrest, and death) [30-32]. Perhaps due to the co-occurrence of other risk factors and access to poisonous substances, addiction in family members increased the mortality risk of poisoned children, which should be taken into account [17,18].

Although recall bias is a limitation of case-control studies, its likelihood was low in the present study due to the use of various information sources (interviews with family members of the poisoned children). Furthermore, the generalizability of the results of hospital-based case-control studies can be a challenge, although this limitation was minimized by selecting 2 major large hospitals (Imam Reza and Ghaem Hospitals) that accounted for roughly 90% of all poisoning cases in the region.

The results of this study showed a clear relationship between the incidence of childhood poisoning and factors such as addiction and the presence of physical or mental illnesses in family members, a history of poisoning, access of children to poisonous substances, the mother’s smoking and employment status, and hyperactivity of the children. Furthermore, respiratory poisoning and addiction in family members were associated with an increased risk of mortality among poisoned children in this study. In general, the incidence and mortality rate of childhood poisoning could be reduced by the implementation of educational programs and interventions.

## Figures and Tables

**Table 1.** Demographic and background information of the subjects in the case and control groups

Variables	Case	Control
Sex		
Male	113 (46.5)	246 (50.3)
Female	130 (53.5)	243 (49.7)
Age (yr)		
<1	39 (16.0)	18 (3.7)
1-4	108 (44.4)	183 (37.4)
4-8	58 (23.9)	146 (29.9)
>8	38 (15.6)	142 (29.0)
Residence		
Urban	168 (69.1)	421 (86.1)
Rural	75 (30.9)	68 (13.9)
Type of poisoning		
Gastrointestinal	213 (87.7)	-
Respiratory	30 (12.3)	-
History of poisoning		
Yes	91 (37.5)	24 (4.9)
No	152 (62.5)	465 (95.1)
History of poisoning in family		
Yes	66 (27.4)	132 (27.2)
No	175 (72.6)	353 (72.8)
Addiction in family		
Yes	76 (31.3)	93 (19.0)
No	167 (68.7)	396 (81.0)
Presence of physical disease or psychological disorder in family		
Yes	48 (19.7)	44 (9.0)
No	195 (80.2)	445 (91.0)
Hyperactivity		
Yes	75 (30.4)	58 (11.9)
No	169 (69.5)	431 (88.1)
Father's smoking status		
Smoker	91 (37.4)	102 (20.9)
Non-smoker	152 (62.5)	387 (79.1)
Mother's smoking status		
Smoker	50 (20.6)	35 (7.2)
Non-smoker	193 (7.2)	454 (92.8)
Mother's occupation		
Housewife	134 (44.8)	379 (77.5)
Employee	109 (55.1)	110 (22.5)
Access to poisonous substances		
Yes	106 (65.8)	46 (9.4)
No	137 (34.2)	443 (90.6)
Outcome of admission		
Discharge or improvement	229 (96.2)	478 (98.0)
Death	9 (3.8)	10 (2.0)
Duration of hospitalization (hr)	29.13	30.86

Values are presented as number (%).

**Table 2.** Risk factors for childhood poisoning by regression analysis

Variables		Univariate		Multiple	
		OR (95% CI)	p-value	OR (95% CI)	p-value
Sex	Male	1.00 (reference)	-	-	-
	Female	0.86 (0.63, 1.16)	0.332	-	-
Age (yr)	<1	8.09 (4.17, 15.71)	<0.001	6.62 (2.95, 15.00)	<0.001
	1-4	2.20 (1.43, 3.38)	<0.001	2.79 (1.62, 4.80)	0.010
	4-8	1.48 (0.92, 2.37)	0.100	0.82 (0.44, 1.51)	0.530
	>8	1.00 (reference)	-	1.00 (reference)	-
Residence	Urban	0.36 (0.25, 0.52)	0.001	-	-
	Rural	1.00 (reference)	-	-	-
Mother’s occupation	Housewife	1.00 (reference)	-	1.00 (reference)	-
	Employee	2.80 (2.01, 3.89)	<0.001	2.96 (1.92, 4.55)	0.010
Mother’s smoking status	Non-smoker	1.00 (reference)	-	1.00 (reference)	-
	Smoker	3.36 (2.11, 5.34)	0.010	3.44 (1.79, 6.59)	0.010
History of poisoning	No	1.00 (reference)	-	1.00 (reference)	
	Yes	11.59 (7.13, 18.85)	0.001	10.44 (5.58, 19.51)	<0.001
Addiction in family	No	1.93 (1.36, 2.75)	0.010	1.56 (1.06, 2.31)	0.010
	Yes	1.00 (reference)	-	1.00 (reference)	-
History of poisoning in family	No	1.01 (0.71, 1.42)	0.962	-	-
	Yes	1.00 (reference)	-	-	-
Hyperactivity	No	1.00 (reference)	-	1.00 (reference)	-
	Yes	3.25 (2.20, 4.79)	<0.001	3.24 (1.93, 5.42)	0.010
Father’s smoking status	Smoker	2.71 (1.60, 3.18)	0.001	-	
	Non-smoker	1.00 (reference)	-	-	
Access to poisonous substances	Yes	7.45 (5.01, 11.06)	0.001	8.88 (5.41, 14.56)	<0.001
	No	1.00 (reference)	-	1.00 (reference)	-
Presence of physical disease or psychological disorder in family	Yes	2.48 (1.59, 3.87)	0.010	2.07 (1.12, 3.81)	0.050
	No	1.00 (reference)	-	1.00 (reference)	-
Outcome of admission	Discharge or improvement	0.53 (0.21, 1.33)	0.176	-	-
	Death	1.00 (reference)	-	-	-
	Duration of hospitalization (hr)	0.10 (0.99, 1.03)	0.436	-	-

OR, odds ratio; CI, confidence interval.

**Table 3.** Risk factors for mortality due to childhood poisoning by multiple logistic regression analysis

Variables	Death	Survival	OR (95% CI)	p-value
Mother’s occupation (employee)	5 (26.3)	212 (30.0)	0.97 (0.25, 3.73)	0.975
Mother’s smoking status	2 (10.5)	83 (11.7)	1.07 (0.21, 5.35)	0.927
History of poisoning	3 (15.8)	112 (15.8)	0.80 (0.19, 3.28)	0.758
Presence of physical disease or psychological disorder in family	8 (42.1)	531 (75.1)	3.36 (0.86, 13.04)	0.070
Hyperactivity	1 (5.3)	131 (18.5)	0.26 (0.03, 2.17)	0.217
Addiction in family	11 (57.9)	158 (22.3)	4.54 (1.10, 18.68)	0.030
Access to poisonous substances	11 (57.9)	565 (79.9)	2.67 (0.65, 10.95)	0.172
Respiratory poisoning	2 (22.2)	20 (8.7)	6.72 (1.40, 32.07)	0.017

Values are presented as number (%). OR, odds ratio; CI, confidence interval.
